# Nitrogen Fixation Aligns with *nifH* Abundance and Expression in Two Coral Trophic Functional Groups

**DOI:** 10.3389/fmicb.2017.01187

**Published:** 2017-06-28

**Authors:** Claudia Pogoreutz, Nils Rädecker, Anny Cárdenas, Astrid Gärdes, Christian Wild, Christian R. Voolstra

**Affiliations:** ^1^Red Sea Research Center, Division of Biological and Environmental Science and Engineering (BESE), King Abdullah University of Science and Technology (KAUST),Thuwal, Saudi Arabia; ^2^Marine Ecology and Coral Reef Ecology Group, Faculty of Biology and Chemistry, University of BremenBremen, Germany; ^3^Department of Ecology, Leibniz Center for Tropical Marine ResearchBremen, Germany; ^4^Tropical Marine Microbiology Group, Department of Biogeochemistry and Geology, Leibniz Center for Tropical Marine ResearchBremen, Germany

**Keywords:** coral reefs, metaorganism, symbiosis, nitrogen cycling, heterotrophy, autotrophy, diazotrophy, bacteria

## Abstract

Microbial nitrogen fixation (diazotrophy) is a functional trait widely associated with tropical reef-building (scleractinian) corals. While the integral role of nitrogen fixation in coral nutrient dynamics is recognized, its ecological significance across different coral functional groups remains yet to be evaluated. Here we set out to compare molecular and physiological patterns of diazotrophy (i.e., *nifH* gene abundance and expression as well as nitrogen fixation rates) in two coral families with contrasting trophic strategies: highly heterotrophic, free-living members of the family Fungiidae (*Pleuractis granulosa*, *Ctenactis echinata*), and mostly autotrophic coral holobionts with low heterotrophic capacity (Pocilloporidae: *Pocillopora verrucosa*, *Stylophora pistillata*). The Fungiidae exhibited low diazotroph abundance (based on *nifH* gene copy numbers) and activity (based on *nifH* gene expression and the absence of detectable nitrogen fixation rates). In contrast, the mostly autotrophic Pocilloporidae exhibited *nifH* gene copy numbers and gene expression two orders of magnitude higher than in the Fungiidae, which coincided with detectable nitrogen fixation activity. Based on these data, we suggest that nitrogen fixation compensates for the low heterotrophic nitrogen uptake in autotrophic corals. Consequently, the ecological importance of diazotrophy in coral holobionts may be determined by the trophic functional group of the host.

## Introduction

Tropical reef-building (scleractinian) corals are holobionts consisting of the coral animal host, dinoflagellate algae of the genus *Symbiodinium*, and a diverse assemblage of other microbes (Rohwer et al., 2002). These microbes form host-specific associations and provide key functional traits within the coral holobiont. Among these traits, biological nitrogen fixation is considered to be of high ecological significance, as nitrogen fixing Bacteria and Archaea, the diazotrophs, are widely associated with corals (Lema et al., 2012, 2014). Indeed, coral-associated nitrogen fixation provides an important source of nitrogen for *Symbiodinium* (Lesser et al., 2007; Benavides et al., 2016; Pogoreutz et al., 2017), thereby helping to sustain holobiont productivity when nutrients are scarce (Cardini et al., 2015).

While nitrogen fixation rates in scleractinian corals exhibit species-specific variation (Shashar et al., 1994; Cardini et al., 2015), the molecular and ecological drivers of these patterns remain unexplored. Scleractinian coral holobionts are mixotrophic, i.e., can draw energy and nutrients from both autotrophic and heterotrophic sources. Importantly, coral holobionts range from being mostly heterotrophic to mostly autotrophic (Rahav et al., 1989; Muscatine, 1990). As nitrogen fixation is a highly energy-consuming functional trait (Mortenson, 1964), its contribution (and thereby relevance) to holobiont metabolism may differ among trophic functional groups of corals. In this study, we therefore set out to compare rates of nitrogen fixation with coral-associated diazotroph abundance and activity in a comparative coral taxonomic framework assaying highly heterotrophic (*Pleuractis granulosa, Ctenactis echinata*) and mostly autotrophic (*Pocillopora verrucosa*, *Stylophora pistillata*) corals (Muscatine et al., 1984; Muscatine and Kaplan, 1994; Houlbrèque and Ferrier-Pagès, 2009; Ziegler et al., 2014). To do this, we indirectly quantified coral holobiont-associated microbial nitrogen fixation activity from acetylene reduction assays (ARA) (Rädecker et al., 2014). In addition, we assessed coral tissue-associated relative gene copy numbers and expression of the *nifH* gene, a common biomarker for diazotrophs (Gaby and Buckley, 2012) using quantitative PCR (qPCR) to investigate relative diazotroph community sizes along with their activity.

## Materials and Methods

### Collection and Maintenance of Corals

Four species of coral, i.e., two species of Fungiidae (*P. granulosa, C. echinata*) and Pocilloporidae (*P. verrucosa*, *S. pistillata*) were collected at the wave-exposed site of the inshore reef Fsar (22° 13.974N, 39° 01.760E) off the Saudi Arabian coastline in the Central Red Sea during February 2016. The Saudi Coastguard Authority under the auspices of KAUST University issued sailing permits to the collection site, which included sampling of coral specimens. The above coral families species were selected due to their distinct trophic ecologies (Alamaru et al., 2009; Hoeksema and Waheed, 2012; Ziegler et al., 2014) and their previous use in physiological and molecular studies (Muscatine and Kaplan, 1994; Bayer et al., 2013; Baria et al., 2015; Roder et al., 2015; Sawall et al., 2015; Röthig et al., 2016; Pogoreutz et al., 2017).

Five replicate samples were collected for each coral species for nitrogen fixation measurements and three replicate samples for RNA/DNA extractions, respectively (i.e., [5 + 3] replicate samples × 4 species = 32 samples). We collected individuals of equal size for Fungiidae and colony fragments for Pocilloporidae. For nucleic acid extraction, all samples were flash-frozen in liquid nitrogen immediately after collection and stored at -80°C until further processing. Coral samples for nitrogen fixation measurements were transferred to the wet lab facility of the Coastal and Marine Resources (CMOR) Core Lab at the King Abdullah University of Science and Technology (KAUST). Corals were acclimated for 4 weeks prior to the start of the experiment in separate flow-through aquarium tanks and continuously supplied with sediment-filtered seawater from inshore reefs located 1.5 km off KAUST to achieve constant maintenance conditions (150 L aquaria, seawater turnover rate 300 L h^-1^, temperature at ∼28°C; salinity at 40; dissolved oxygen > 6.0 mg O_2_ l^-1^, dissolved inorganic nitrogen ≤ 0.6 μMol l^-1^, and phosphorus ≤ 0.3 μMol l^-1^ at all times; photon flux of ∼150 μmol m^-2^ s^-1^ on a 12:12 h light/dark cycle).

### Nitrogen Fixation Measurements

**FIGURE 1:**
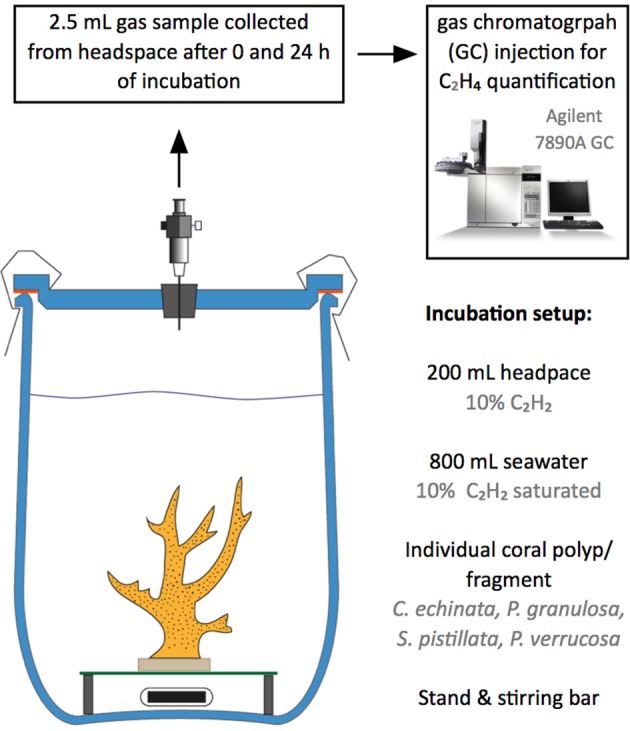
Overview of the incubation setup for the indirect quantification of nitrogen fixation rates of corals using the acetylene (C_2_H_2_) reduction assay (ARA). Individual coral fragments were incubated in 1 L incubation jars enriched with C_2_H_2_. Gas samples were collected at the start and end of incubation for ethylene (C_2_H_4_) quantification using gas chromatography.

Nitrogen fixation rates were calculated indirectly from ethylene (C_2_H_4_) evolution via ARA incubations as described previously (Rädecker et al., 2014; Pogoreutz et al., 2017). Incubations were conducted in gas-tight 1 L glass chambers in 800 ml of seawater and 200 ml of 20% acetylene-enriched air headspace (**Figure 1**). Each jar contained a single coral sample (i.e., individual polyp or colony fragment, see above). Five coral samples per species were incubated in seawater. Two additional chambers filled exclusively with seawater (i.e., without coral samples) served as controls to correct for planktonic background metabolism. During the 24 h incubations, chambers were submersed in a tempered water bath and constantly stirred (600 rpm) to ensure stable measurement conditions (28°C, 12:12 h light/dark cycle, photon flux of ∼150 mol μmol m^-2^ s^-1^). Gas samples were collected after 0 and 24 h of incubation. C_2_H_4_ concentrations in gas samples were quantified by gas chromatography and flame ionization detection (Agilent 7890A GC system with Agilent HP-AL/S column (Agilent technologies, United States); lower detection limit for C_2_H_4_ = 1 ppm). Ethylene concentrations were normalized to coral surface areas, which were based on 3D models of the coral skeleton generated with the software Remake v117.25.67 (Autodesk Inc., United States). As we acknowledge the ongoing discussion regarding the appropriate conversion factor, nitrogen fixation is presented as C_2_H_4_ evolution rates (mean ± SE) without conversion into actual nitrogen fixation rates (Hardy et al., 1968; Wilson et al., 2012).

### Nucleic Acid Isolation and qPCR

Tissue-associated relative gene copy numbers and expression of the *nifH* gene were quantified for each coral species using a qPCR assay. For this purpose, RNA and DNA were extracted from all corals using the Qiagen AllPrep DNA/RNA Mini Kit (Qiagen, Germany) according to manufacturer’s instructions. Coral tissue was air-blasted off the skeleton on ice with RLT Plus buffer and using airflow from a sterile air gun. For *P*. *granulosa* and *C. echinata*, tissue was blasted off from both oral (top) and aboral (bottom) surfaces and pooled subsequently. A total of 600 μl tissue slurry per sample were used for RNA and DNA extractions. DNA extractions were quantified and quality checked using a NanoDrop 2000c (Thermo Scientific, United States) and adjusted to a concentration of 10 ng μL^-1^. For cDNA synthesis, the SuperScript III First Strand Synthesis SuperMix kit (Thermo Scientific, United States) was used according to manufacturer’s instructions using 500 ng of total RNA input in 20 μL reactions.

The qPCRs were run in triplicates for gDNA (to quantify gene copy number) and cDNA (to quantify gene expression) with the Platinum SYBR Green qPCR SuperMix kit (Invitrogen, Carlsbad, CA, United States) using 5 μL SuperMix, 0.2 μL ROX reference dye, 0.2 μL of each 10 μM primer, 1 μL of cDNA, and RNAse-free water to adjust the reaction volume to 10 μl. Determination of relative gene copy numbers (from DNA) and mRNA expression of the *nifH* gene in coral tissue was determined by normalization against the ITS2 of *Symbiodinium* (Arif et al., 2014). This multi copy gene marker is exceptionally well-suited for this purpose given its high abundance, stable expression, and the circumstance that *Symbiodinium* show a constant cell-specific density in the majority of coral species (Muscatine et al., 1998). Of note, *Symbiodinium* are the most important sink for fixed nitrogen in the coral holobiont (Falkowski et al., 1993; Lesser et al., 2007; Cardini et al., 2015; Aranda et al., 2016; Pogoreutz et al., 2017). For the amplification of *nifH*, the primers F2 5′-TGYGAYCCIAAIGCIGA-3′ and R6 5′-TCIGGIGARATGATGGC-3′ were used (Gaby and Buckley, 2012). To amplify the *Symbiodinium* ITS2 region, the primers ITSintfor2 5′-GAATTGCAGAACTCCGTG-3′ and ITS2-reverse 5′-GGGATCCATATGCTTAAGTTCAGCGGGT-3′ were used (LaJeunesse, 2002). All amplicons were amplified in the same qPCR run using the following thermal profile: 2 min at 50°C, 1 min at 94°C, followed by 50 cycles of 94°C for 30 s, 51°C for 1 min, 72°C for 1 min, and an extension cycle of 1 min at 72°C. Specificity of amplification was confirmed by melting curve analysis. Standard calibration curves were run simultaneously covering 6 orders of magnitude (10^4^–10^9^ copies of template per assay). Relative fold change of copies of the *nifH* gene was calculated as 2^(-ΔΔCt)^ against ITS2 gene copy numbers using *P. granulosa* samples as the reference.

### Statistical Analysis

All statistical analyses were run in SigmaPlot 13 (Systat Software GmbH, Germany). A student’s *t*-test was run to assess species level differences on nitrogen fixation rates. To assess family level-differences in *nifH* gene copy numbers and expression, a one-way ANOVA was conducted with subsequent *post hoc* Bonferroni Correction. To test for a significant relationship between *nifH* gene copy number and expression within each of the two coral families, a non-linear regression was conducted with relative gene copy numbers as the predictor and relative gene expression as the dependent variable.

## Results

**FIGURE 2:**
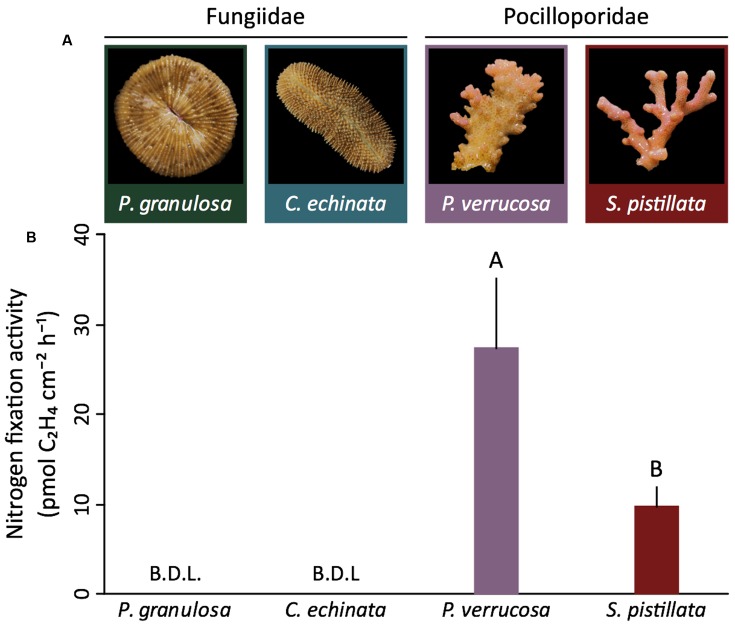
Microbial nitrogen fixation rates in four tropical scleractinian corals from two different trophic functional groups. **(A)** Representative specimens of the investigated coral species from the highly heterotrophic Fungiidae and the mostly autotrophic Pocilloporidae. **(B)** Coral-associated nitrogen fixation rates (*n* = 5 per species) as assessed indirectly from acetylene reduction assays. Nitrogen fixation rates of the pocilloporids *P. verrucosa* and *S. pistillata* are significantly different from each other (*t*-test, *t* = 3.17, *p* = 0.01). In contrast, nitrogen fixation remained below the detection limit (B.D.L.) in both Fungiidae. Nitrogen fixation rates are averaged over 24 h (i.e., include light and dark fixation) and presented as mean ± SE. Different letters above bars indicate significant differences between groups (*p* < 0.05).

**FIGURE 3:**
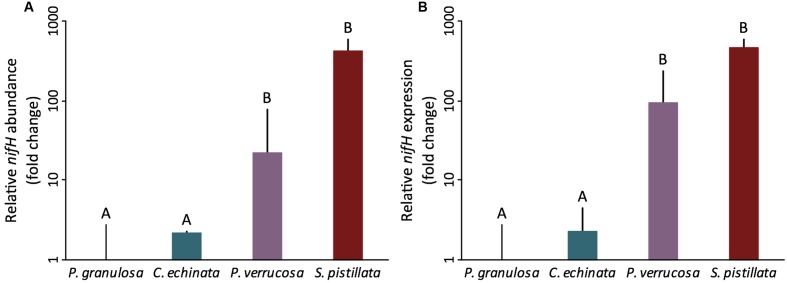
Diazotroph abundance **(A)** and activity **(B)** in four tropical scleractinian corals (*n* = 3 per species) from two different trophic functional groups (the highly heterotrophic Fungiidae and the mostly autotrophic Pocilloporidae) as measured by coral-tissue associated *nifH* relative gene copy numbers and expression referenced against the Internal Transcriber Space 2 (ITS2) of *Symbiodinium* via qPCR. Significant family-level differences are apparent for relative *nifH* gene copy numbers and gene expression (ANOVA, *F* = 40.61 *p* < 0.001 and ANOVA, *F* = 12.30, *p* < 0.001, respectively). Data are shown as mean fold changes in relation to *P. granulosa*; error bars indicate upper confidence interval. Different letters above bars indicate significant differences between groups (*p* < 0.05).

Both species of the mostly autotrophic Pocilloporidae (*P. verrucosa*, *S. pistillata*) exhibited detectable gross (i.e., holobiont) nitrogen fixation (0.03 ± 0.04 and 0.01 ± 0.00 nmol C_2_H_4_ day^-1^ cm^-2^, respectively) (**Figures 2A,B**). In contrast, nitrogen fixation remained below the detection limit for both species (*P. granulosa, C. echinata*) of the highly heterotrophic Fungiidae (**Figures 2A,B**). This pattern of differential nitrogen fixation activity between the two coral families aligned with *nifH* abundance and expression. Corals in the Pocilloporidae family showed a significantly higher abundance of nitrogen-fixing bacteria (assessed via relative *nifH* gene copy number) compared to both members of the Fungiidae family (ANOVA, *F* = 40.61, *p* < 0.001; **Figure 3A** and Supplementary Table S1). A similar pattern was confirmed for diazotroph activity, with the Pocilloporidae having higher relative *nifH* gene expression than the two fungiid species (ANOVA, *F* = 12.30, *p* < 0.001; **Figure 3B** and Supplementary Table S1). Specifically, *P. verrucosa* and *S. pistillata* showed 23- and 431-fold higher relative diazotroph abundance and 95- to 480-fold higher relative gene expression compared to *P. granulosa*, respectively (Supplementary Table S1). Non-linear regression revealed a strong significant relationship between *nifH* copy numbers and expression in the two species of Pocilloporidae (*R*^2^ = 71.49, *p* = 0.0339), but only a weak although significant relationship in the two Fungiidae (*R*^2^ = 0.07, *p* = 0.0288).

## Discussion

The present study investigated physiological and molecular patterns of nitrogen fixation along with diazotroph abundance in corals representing two different trophic functional groups (autotrophy vs. heterotrophy). This was achieved by assessing nitrogen fixation activity (as assessed via ARA) as well as tissue-associated relative gene copy numbers and expression of the *nifH* gene (as assessed via qPCR) in a comparative species framework. The investigated corals exhibited similar patterns within functional groups, but marked differences between functional groups for all measurements.

Our data are within the range of nitrogen fixation rates published for pocilloporid corals (Shashar et al., 1994; Rädecker et al., 2014; Cardini et al., 2015; Pogoreutz et al., 2017). Similarly, an absence of detectable nitrogen fixation was previously reported for the fungiid coral *Fungia fungites* (Shashar et al., 1994). Our present study shows that physiological differences in holobiont nitrogen fixation rates align with relative *nifH* gene abundance and activity, and hence, likely reflect differences of nitrogen-fixing bacteria in the tissues of these corals, highlighting molecular differences on the coral family level.

To understand the distinct physiological and molecular patterns in diazotrophy, the ecological context of the investigated coral families, particularly the trophic functional groups, must be considered. The observed patterns in diazotrophy align with differential heterotrophic feeding capacities of the investigated coral holobionts. While Fungiidae are highly heterotrophic and can exploit a large variety of food sources (Houlbrèque and Ferrier-Pagès, 2009), shallow-water Pocilloporidae have a very limited heterotrophic capacity and strongly rely on photoautotrophy (Muscatine et al., 1984; Muscatine and Kaplan, 1994; Ziegler et al., 2014). Given that the investigated functional groups align with family association, it remains to be determined whether the observed differences are reflecting ecological differences in autotrophic and heterotrophic corals at large, e.g., by the incorporation of further species. In any case, photosynthetically fixed carbon (photosynthate) is characterized by high C:N ratios compared to heterotrophic food sources (Falkowski et al., 1984; Muscatine et al., 1984). Therefore, high carbon availability coupled with strong nitrogen limitation in mostly autotrophic coral holobionts may select for relatively larger diazotroph populations compared to mostly heterotrophic coral holobionts. These results suggest that the importance of microbial nitrogen fixation depends on the heterotrophic capacity of the coral holobiont. This view is supported by previous studies showing differential partitioning of δ^15^N in corals along a water depth gradient, indicating a higher importance of nitrogen fixation products in low-light adapted phototrophs compared to heterotrophs (Muscatine and Kaplan, 1994; Bednarz et al., 2017).

The observed physiological and molecular patterns in nitrogen fixation have important implications for our understanding of holobiont nutrient cycling and functioning. For instance, changes in nutrient cycling and stoichiometry can be involved in coral symbiotic breakdown as recently shown (Wiedenmann et al., 2012; Ezzat et al., 2016; Pogoreutz et al., 2017). Indeed, while the input of ‘new’ nitrogen from diazotrophy may be beneficial for holobiont functioning in oligotrophic reef waters (Cardini et al., 2015; Benavides et al., 2016), stimulated nitrogen fixation activity may be linked to detrimental effects on *Symbiodinium* photophysiology and coral bleaching (Santos et al., 2014; Rädecker et al., 2015; Pogoreutz et al., 2017). Hence, diazotrophs may be considered beneficial microorganisms for corals (BMCs) under stable, oligotrophic conditions (Peixoto et al., 2017). Yet, their presence may affect the corals’ susceptibility to stress during environmental change highlighting the importance of understanding the role of microbes in holobiont functioning and breakdown (Sweet and Bulling, 2017). Indeed, Pocilloporidae are highly sensitive to thermal stress (McClanahan, 2004; Pinzón et al., 2013; Swain et al., 2016), whereas Fungiidae are resistant to a range of environmental stressors (Chadwick and Loya, 1990; Hoeksema, 1991; Philipp and Fabricius, 2003; Baria et al., 2015; Röthig et al., 2016; Swain et al., 2016). We thus argue that a strong coral host dependency on microbial nitrogen fixation may render corals more vulnerable to the effects of environmental change. Hence, future research efforts should be directed to disentangle a potential link between coral-associated nitrogen fixation and holobiont stress susceptibility. Understanding this link will assist to improve current reef conservation measures.

Taken together, our study provides insight into the ecological underpinnings that may underlie differences in diazotrophy of the coral holobiont with implications for coral host environmental resilience. As such, our work contributes to the growing body of literature that suggests the importance of microbes to metaorganism function. Future work assessing the diversity and composition of coral-associated diazotroph communities in a comparative coral taxonomic framework, e.g., via *nifH* gene sequencing (Lema et al., 2012; Santos et al., 2014) will provide additional insight into host-microbe interactions.

## Author Contributions

CP, NR, CW, and CV conceived and designed the experiment. CP and NR conducted the experiments, analyzed, and interpreted the data. AC, CW, and CV contributed reagents and tools. CP, NR, CW, and CV drafted and all authors revised the article. All authors have agreed to and approved the final article.

## Conflict of Interest Statement

The authors declare that the research was conducted in the absence of any commercial or financial relationships that could be construed as a potential conflict of interest.
