# Supplementary material for: Nitrogen Fixation Aligns with nifH Abundance and Expression in Two Coral Trophic Functional Groups
Source: Front Microbiol. 2017 Jun 28;8:1187. doi: 10.3389/fmicb.2017.01187 (PMC5487474; doi:10.3389/fmicb.2017.01187)
Supplement: Supplementary file 1 [file Table_1.DOCX]

**Supplementary Material**

**Table S1.** Ct values from qPCR reactions representing gene abundance (gDNA) and expression (cDNA) of the *nifH* and the ITS2 gene for the four coral species used in this study. Data are shown as mean ± SE.

|  | Gene abundance | | Gene expression | |
| --- | --- | --- | --- | --- |
|  | Ct *nifH* | Ct ITS2 | Ct *nifH* | Ct ITS2 |
| *P. granulosa* | 31.55 ± 0.67 | 18.28 ± 1.06 | 43.73 ± 1.78 | 22.61 ± 0.69 |
| *C. echinata* | 32.35 ± 0.58 | 20.20 ± 0.63 | 42.58 ± 0.41 | 22.65 ± 0.94 |
| *P. verrucosa* | 30.15 ± 2.21 | 21.41 ± 0.83 | 40.53 ± 0.97 | 25.99 ± 1.74 |
| *S. pistillata* | 26.67 ± 0.53 | 22.26 ± 0.73 | 36.10 ± 0.85 | 23.89 ± 0.68 |
